# Comparative analysis of target volume coverage and liver exposure in high-dose-rate interstitial brachytherapy and *in silico* MR LINAC-based stereotactic body radiotherapy plans for colorectal liver metastases

**DOI:** 10.1016/j.ctro.2025.101030

**Published:** 2025-08-11

**Authors:** Sina Mansoorian, Svenja Hering, Jan Hofmaier, Yuqing Xiong, Helmut Weingandt, Maya Rottler, Franziska Walter, Paul Rogowski, Max Seidensticker, Jens Ricke, Claus Belka, Stefanie Corradini, Chukwuka Eze

**Affiliations:** a Department of Radiation Oncology, LMU University Hospital, LMU Munich, Munich, Germany; b Department of Radiology, LMU University Hospital, LMU Munich, Munich, Germany; c German Cancer Consortium (DKTK), partner site Munich and German Cancer Research Center (DKFZ), Heidelberg, Germany; d Bavarian Cancer Research Center (BZKF), Munich, Germany

**Keywords:** Colorectal liver metastasis, CT-guided interstitial brachytherapy, Dosimetry, HDR interstitial brachytherapy, In silico analysis, SABR, SBRT, Stereotactic ablative radiotherapy

## Abstract

**Background:**

This study compared the plan quality and dosimetric parameters of single-fraction (SF) MR-LINAC (MRL)-based stereotactic body radiotherapy (SBRT) with delivered high-dose-rate interstitial brachytherapy (HDR-iBT) for colorectal liver metastases (CRLM).

**Methods:**

Between August 2017 and March 2019, 26 patients with a total of 45 CRLM were treated in 28 sessions using HDR-iBT with 1 × 25 Gy and were retrospectively included in this study. For each patient, an *in silico* MRL-based SBRT plan was generated using the corresponding iBT CT dataset. In the iBT plans, a single fraction of 25 Gy was prescribed to the periphery of the gross tumor volumes (GTVs), while in the SBRT plans, the same dose was prescribed to the 80% isodose line covering the planning target volumes (PTVs). We compared the dosimetric properties of the delivered HDR-iBT and MRL-based SBRT plans.

**Results:**

Median GTV was 3.83 cc (range: 0.13–92.58 cc) and median PTV_SBRT_ was 15.47 cc (range: 2.68–164.17 cc). Both HDR-iBT and SBRT demonstrated excellent GTV coverage, with no statistically significant differences in GTV D_98%_ (28.82 ± 2.57 Gy vs. 28.92 ± 0.88 Gy, p = 0.9). HDR-iBT achieved superior GTV D_95%_ (31.62 ± 3.20 Gy vs. 29.22 ± 0.74 Gy, p < 0.01) and GTV D_50%_ (64.71 ± 12.78 Gy vs. 30.22 ± 0.52 Gy, p < 0.01). Uninvolved liver dose metrics were higher in the SBRT plans compared to iBT, with a median relative difference in V_5Gy_ of 5.29% (range: −13.69% to + 17.89%, p < 0.01) and a smaller relative difference in V_10Gy_ of 1.5% (range: −7.74% to + 11.26%, p < 0.01).

**Conclusion:**

Our comparison indicates MRL-based SBRT to liver lesions is feasible, achieving adequate target volume coverage without clinically relevant violations of organ-at-risk (OAR) constraints.

## Highlights

• Target Coverage and Dose metrics: Both HDR-iBT and SBRT provided excellent target coverage with similar GTV D_98%_ values.
• HDR-iBT showed higher doses based on GTV D_95%_ and D_50%_.
• SBRT provided consistent and reliable dose delivery, reducing variability compared to HDR-iBT.
Both modalities adhered to clinical OAR dose constraints, with acceptable uninvolved liver exposure, supporting the feasibility of MRL-SBRT.

## Introduction

Colorectal liver metastases (CRLM) are frequently managed with various local treatment modalities, including ablation, embolization, stereotactic body radiotherapy (SBRT), and high-dose-rate (HDR) interstitial brachytherapy (iBT) [[1], [2], [3]]. Studies have reported on interstitial brachytherapy (iBT) in managing liver metastases in the oligometastatic setting [4]. Furthermore, It has been shown that improved target volume dose coverage and reduced uninvolved liver exposure with HDR-iBT vs. multi-fraction LINAC-based SBRT in hepatocellular carcinoma [5].

Real-time MR-guided SBRT (MRgSBRT) is a relatively novel treatment technique that allows for superior tumor visualization, anatomical plan adaptation, and continuous tumor gating. Initial studies indicate excellent target volume coverage and sparing of organs at risk (OARs) across different tumor types, including primary and secondary liver cancers [[6], [7], [8]]. However, there is a lack of data vis-à-vis plan quality and dosimetric properties of HDR-iBT vs. single fraction (SF-)SBRT, with particular emphasis on managing multiple liver lesions (treated with a single plan) and differences in uninvolved liver exposure. Thus, in the current study, we compared the plan quality and dosimetric parameters of *in silico* single-fraction SBRT plans to those of delivered HDR-iBT plans for colorectal liver metastases.

## Material/Methods

From August 2017 to March 2019, 26 patients with a total of 45 colorectal liver metastases underwent treatment with a 1 × 25 Gy HDR-iBT, delivered over 28 sessions, at a tertiary academic center. These patients were retrospectively included in the study based on predefined criteria, which required the presence of metastatic colorectal liver metastases. Before treatment, all patients gave informed consent to use their anonymized data for research purposes (Ethics approval reference number: LMU-18-511).

During the brachytherapy procedure, catheters were placed by an experienced interventional radiologist using an expiration breath-hold technique, with imaging performed at a slice thickness of 2 mm for precision. The gross tumor volume (GTV) was outlined by a radiation oncologist with expertise in brachytherapy, who utilized diagnostic liver MRI scans with hepatocyte-specific contrast agents and/or contrast-enhanced CT scans to ensure accurate tumor delineation.

For each patient, an *in silico* MR LINAC (MRL)-based SBRT plan was generated using the corresponding simulation CT datasets and anatomical structures used for brachytherapy on the MRIdian system (previously ViewRay Inc., presently ViewRay Systems, USA) treatment planning system (TPS). For the SBRT plans, the planning target volumes (PTV_SBRT_) were created by expanding the GTV isotropically by 5 mm. The specifics of the HDR-iBT procedure have been thoroughly described in our previous publications [[4], [5]]. The simulation CT datasets were exported from the Oncentra brachytherapy planning system (Elekta AB, Stockholm, Sweden, version 4.5.2). This software calculates doses based on the AAPM TG-43U1 formalism. For the HDR-iBT plans, a prescription dose of 25 Gy was delivered directly to the GTVs. The CT datasets were then imported into the MRIdian TPS. Similarly, the SBRT plans were designed to deliver a single dose of 25 Gy, prescribed to the 80% isodose line (IDL) covering the PTVs. All plans were optimized to ensure that 98 % of the PTV received 100% of the prescribed dose (PTV D_98%_ = prescribed dose).

We evaluated the dosimetric properties of delivered HDR-iBT vs. *in silico* SBRT plans, focusing on the coverage of GTV_BT/SBRT_ and PTV_SBRT_, the OARs, and the exposure to uninvolved liver, including the available single-fraction liver dose constraints across multiple publications [[9], [10], [11], [12]]. Dosimetric parameters were extracted from the Oncentra brachytherapy planning system for iBT plans and the MRIdian TPS for *in silico* SBRT plans. The percentage of GTV/PTV receiving the prescribed dose (V_25Gy_), the mean GTV doses (GTV D_mean_), and the minimum doses to 2%, 50%, 95%, 98% and 100% of the PTV/GTV (PTV/GTV D_2%_ = near maximum dose, PTV/GTV D_50%_ = median dose, PTV/GTV D_95%_, PTV/GTV D_98%_ = near minimum dose, GTV D_100%_ = minimum dose) were extracted from the TPS.

The conformity index (CI) is defined as PIV/PTV, where PIV is the prescription isodose volume and PTV is the planning target volume. The biologically effective dose (BED) is determined using the formula: BED = n × d × [1 + d / (α/β)], where “n” represents the number of fractions, “d” is the dose per fraction, and “α/β” denotes the tissue's sensitivity to radiation. For tumor, the α/β value is typically considered to be 10 Gy. To reflect this, we define BED_10_ as the BED calculated with α/β = 10 Gy, which is typically applied in tumor response modeling. The prescribed single-fraction dose of 25 Gy at the periphery corresponds to a BED_10_ of 87.5 Gy.

The Wilcoxon signed-rank test was conducted to compare dosimetric parameters. A p-value of less than 0.05 was deemed statistically significant. Statistical analyses were conducted using IBM SPSS Statistics software (version 29.0.1) and GraphPad Prism (version 10.5.0 for Windows, GraphPad Software, Boston, Massachusetts, USA).

## Results

Patient and treatment characteristics are presented in Table 1.

**Table 1:** Patient and lesion characteristics.

Characteristics	median (range)	N (%)
Total patients		26 (100%)
Sex		
Male		16 (61.5%)
Female		10 (38.5%)
Age [years]		
median (range)	65 (32–87)	
Primary tumor		
Rectosigmoid cancer		19 (73.1%)
Colon cancer		7 (26.9%)
Total treated metastases		45 (100%)
Lesion count per treatment session		
1		18 (64.3%)
2		5 (17.9%)
3		4 (14.3%)
4		0 (0.0%)
5		1 (3.6%)
GTV_BT_		
median (range) [cc]	3.83 (0.13–92.58)	
PTV_SBRT_		
median (range) [cc]	15.47 (2.68–164.17)	

GTV_BT_; gross tumor volume for brachytherapy; PTV_SBRT_; planning target volume for stereotactic body radiotherapy.

## Dosimetric comparison of *in silico* SBRT vs. iBT plans

**Fig. 1:**
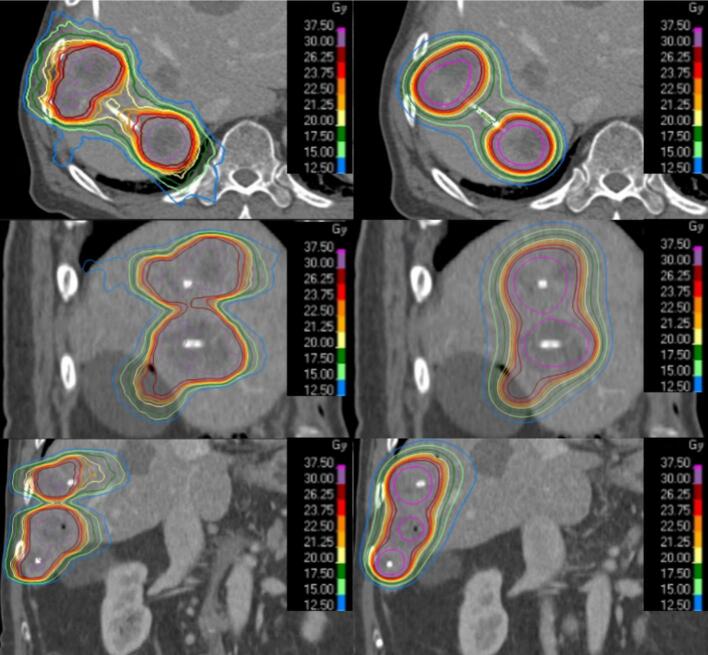
Comparison of isodose distributions in a representative patient: *in silico* SBRT plan (left) vs. brachytherapy plan (right) in axial, sagittal, and coronal views.

*In silico* single-fraction MRL-based SBRT plans were generated. Dose distributions for an exemplary patient with 5 metastases are shown in Fig. 1.

**Fig. 2:**
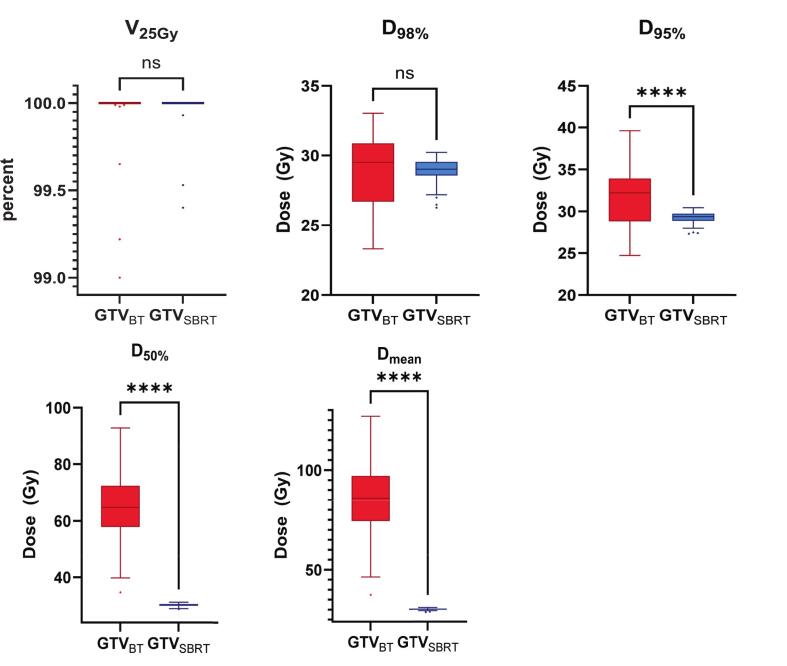
Comparison of GTV dose metrics between brachytherapy (GTV_BT_, red) and stereotactic body radiotherapy (GTV_SBRT_, blue). Box plots display values for V_25Gy_ (percentage of GTV receiving ≥ 25 Gy), D_98%_, D_95%_, D_50%_, and D_mean_. Differences in V_25Gy_ and D_98%_ between BT and SBRT were not statistically significant (ns). In contrast, BT achieved significantly higher D_95%_, D_50%_, and D_mean_ values than SBRT (p < 0.01). Boxes indicate the interquartile range with the median line; whiskers represent the full data range, and individual points show outliers. (For interpretation of the references to colour in this figure legend, the reader is referred to the web version of this article.)

**Fig. 3:**
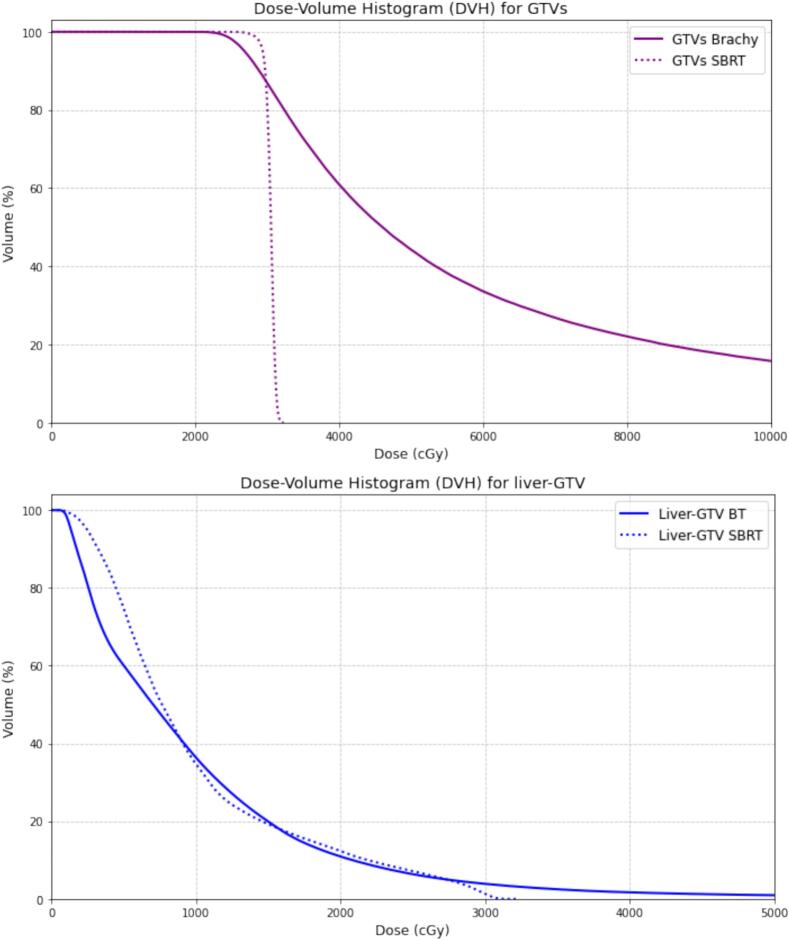
Dose–volume histograms (DVHs) comparing dose distribution to gross tumor volumes (GTVs, top panel) and uninvolved liver (Liver–GTV, bottom panel) in a single patient treated with interstitial brachytherapy (iBT) versus the *in silico* stereotactic body radiotherapy (SBRT) plan. Solid lines represent iBT and dashed lines represent SBRT.

The median GTV was 3.83 cc (range: 0.13–92.58), and the median PTV_SBRT_ was 15.47 cc (range: 2.68–164.17). The mean GTV D_98%_ for iBT and SBRT plans was 28.82 ± 2.57 Gy vs. 28.92 ± 0.88 Gy (p = 0.9), and the mean GTV V_25Gy_ was 99.95 ± 0.19% in iBT plans vs. 99.97 ± 0.11% in SBRT plans (p = 0.6) (Table 2 and Fig. 2, Fig. 3). The mean GTV D_95%_ in iBT vs. SBRT plans was 31.62 ± 3.20 Gy vs. 29.22 ± 0.74 Gy (p < 0.01), and the mean GTV D_50%_ was 64.71 ± 12.78 Gy vs. 30.22 ± 0.52 Gy (p < 0.01). An exemplary dose-volume histogram (DVH) illustrating the comparison of V_25Gy_, D_98%_, and D_50%_ is shown in Fig. 3.

**Table 2:** Dosimetric comparison of HDR-iBT and MRL-based SBRT for target volumes and uninvolved liver.

DVH parameter		iBT	SBRT	p-value	iBT vs. SBRT
		Mean ± SD	Mean ± SD		Δ Median (range)
GTV	V_25Gy_ [%]	99.95 ± 0.19	99.97 ± 0.11	0.6	0.00 (−1.00 to 0.60)
	D_100%_ [Gy]	24.29 ± 1.88	27.97 ± 1.66	**< 0.01**	−3.33 (−4.41 to −2.91)
	D_98%_ [Gy]	28.82 ± 2.57	28.92 ± 0.88	0.9	0.35 (−6.56 to 4.59)
	D_95%_ [Gy]	31.62 ± 3.20	29.22 ± 0.74	**< 0.01**	2.76 (−5.48 to 11.02)
	D_50%_ [Gy]	64.71 ± 12.78	30.22 ± 0.52	**< 0.01**	34.12 (4.02 to 64.13)
	D_mean_ [Gy]	85.72 ± 19.66	30.22 ± 0.48	**< 0.01**	56.28 (6.86 to 97.29)
	D_2%_ [Gy]	Not reported	31.1 ± 0.5		
	D_1cc_ [Gy]	67.50 ± 21.70	30.67 ± 0.60	**< 0.01**	37.27 (3.06 to 64.34)
	CI_GTV_	1.00 ± 0.00	1.00 ± 0.00	0.6	0.00 (0.00 to 0.01)
	HTCI	0.29 ± 0.16	0.31 ± 0.12	0.2	−0.03 (−0.21 to 0.21)
	CN	0.29 ± 0.16	0.31 ± 0.12	0.2	−0.03 (−0.21 to 0.21)
PTV					
	D_98%_ [Gy]		25.03 ± 0.26		
	D_95%_ [Gy]		25.75 ± 0.32		
	D_50%_ [Gy]		29.13 ± 0.46		
	D_2%_ [Gy]		30.95 ± 0.41		
	CI		0.98 ± 0.006		
		**Mean Volume ± SD**	**Mean Volume ± SD**	**p-value**	**iBT vs. SBRT**
					**Δ median (range)**
Uninvolved Liver					
	V_5Gy_ [%]	23.84 ± 18.04	28.05 ± 16.65	**< 0.01**	−5.29 (−17.89 to 13.69)
	V_5Gy_ [cc]	315.91 ± 233.77	381.42 ± 239.25	**< 0.01**	−83.65 (−314.29 to 136.38)
	V_9.1Gy_ [%]	12.17 ± 10.88	14.79 ± 10.85	**< 0.01**	−1.51 (−12.56 to 6.25)
	V_9.1Gy_ [cc]	161.03 ± 139.58	200.11 ± 152.78	**< 0.01**	−18.05 (−219.55 to 62.25)
	V_10Gy_ [%]	10.77 ± 9.80	12.86 ± 9.58	**< 0.01**	−1.51 (−11.26 to 7.74)
	V_10Gy_ [cc]	142.59 ± 125.59	173.79 ± 134.24	**< 0.01**	−18.33 (−219.55 to 62.25)
	V_11Gy_ [%]	9.49 ± 8.75	11.07 ± 8.32	**< 0.01**	−1.12 (−9.69 to 8.79)
	V_11Gy_ [cc]	125.63 ± 111.92	149.53 ± 116.16	**< 0.01**	−17.27 (−169.42 to 95.67)
	V_11.6Gy_ [%]	8.82 ± 8.18	10.13 ± 7.61	**< 0.01**	−0.95 (−9.15 to 8.86)
	V_11.6Gy_ [cc]	116.69 ± 104.47	136.78 ± 105.99	**< 0.01**	−15.05 (−154.78 to 100.75)

Significant p-values marked in bold, DVH – Dose–Volume Histogram; iBT – Interstitial Brachytherapy; SBRT – Stereotactic Body Radiotherapy; GTV – Gross Tumor Volume; PTV – Planning Target Volume; CI – Conformity Index; HTCI – Healthy Tissue Conformity Index; CN – Conformation Number; Gy – Gray; SD – Standard Deviation; Δ Median – Median difference between iBT and SBRT.

When comparing D_100%_ as a surrogate for D_min_ across 45 matched cases, SBRT showed superior GTV coverage in 43 cases (95.56%). In 2 cases (4.44%), SBRT had a less favorable dose profile. Notably, SBRT resulted in D_100%_ values below the prescription dose in 3 cases (6.67%), while this occurred in 17 cases (40%) in the HDR-iBT group. (Supplementary Fig. 1 and Supplementary Table 2).

When comparing the 45 paired treatment plans, GTV coverage by the prescription dose (V_25Gy_ = 100%) was achieved in 42 SBRT plans and 39 iBT plans. Among the remaining SBRT cases, V_25Gy_ ranged from 99.40% to 99.93%. In the iBT group, six plans did not reach V_25Gy_ = 100%, with V_25Gy_ values ranging from 99.00% to 99.99%. (Supplementary Table 2).

Modified indices [13], including the Conformity indices (CI_GTV_) for a more valid comparison of both modalities, were calculated using the GTV instead of PTV, defined as

• **GTV Conformity Index (CI_GTV_)** = V_GTV_PD_/V_GTV_ (0 ≤ CI_GTV_ ≤ 1; ideally 1)
  o Definition: proportion of GTV receiving at least the prescribed dose (PD)
  o V_GTV_PD_: volume of GTV covered with the PD
  o V_GTV_: volume of the GTV

were similar between modalities (*p* = 0.6).

The Homogeneity-Target Conformity Index (HTCI) and Conformation Number (CN):

• **Healthy Tissue Conformity Index (HTCI) =** V_GTV_PD_/V_PD_ (0 ≤ HTCI ≤ 1; ideally 1)
  o Definition: irradiation of healthy tissue beyond the GTV border with the prescribed dose.
  o V_PD_: total volume receiving the prescribed dose
• **Conformation Number (CN) =** CI_GTV_ x HTCI =(V_GTV_PD_)^2^/V_GTV_ x V_PD_ (0 ≤ CN ≤ 1; ideally 1).

also showed no statistically significant differences (both *p* = 0.2).

## Uninvolved liver exposure

**Fig. 4:**
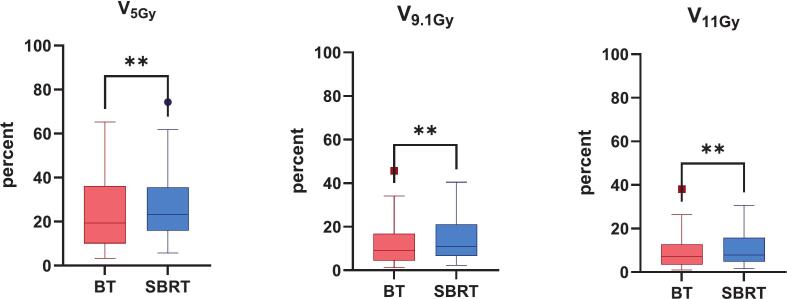
Dose distribution in Uninvolved Liver for V_5Gy_, V_9.1Gy_, V_11Gy_ for interstitial brachytherapy (iBT) and stereotactic body radiotherapy (SBRT). Box plots show the percentage of uninvolved liver volume receiving at least 5  Gy, 9.1  Gy, and 11  Gy. BT values are shown in red, and SBRT values in blue. Across all dose levels, BT resulted in significantly lower exposure of uninvolved liver compared to SBRT (p < 0.01). (For interpretation of the references to colour in this figure legend, the reader is referred to the web version of this article.)

**Fig. 5:**
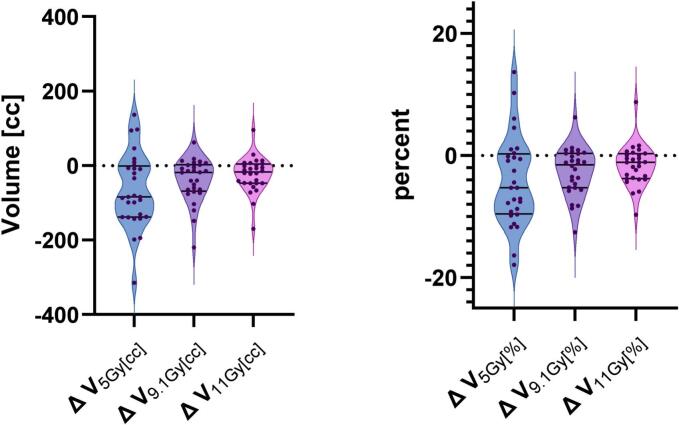
Relative (left panel) and absolute (right panel) differences in liver–GTV dose exposure between interstitial brachytherapy (iBT) and stereotactic body radiotherapy (SBRT). Violin plots depict patient-level differences in liver–GTV volumes at the 5  Gy, 9.1  Gy, and 11  Gy isodose levels. Positive values indicate higher dose exposure with BT, and negative values indicate higher exposure with SBRT. Each plot shows the distribution of individual differences, with the median and interquartile range indicated. The width of the violin reflects the density of data points at each value.

For V_5Gy_, the mean relative volume of uninvolved liver was 23.84 ± 18.04% for iBT and 28.05 ± 16.65% for SBRT (p < 0.01), with corresponding absolute volumes of 315.91 ± 233.77 cc and 381.42 ± 239.25 cc, respectively. At V_9.1Gy_, the relative volumes were 12.17 ± 10.88% for iBT and 14.79 ± 10.85% for SBRT (p < 0.01), with absolute values of 161.03 ± 139.58 cc and 200.11 ± 152.78 cc. Similarly, for V_10Gy_, the relative volumes were 10.77 ± 9.80% for iBT and 12.86 ± 9.58% for SBRT (p < 0.01); absolute volumes were 142.59 ± 125.59 cc and 173.79 ± 134.24 cc. At V_11Gy_, the relative volumes were 9.49 ± 8.75% for iBT and 11.07 ± 8.32% for SBRT (p < 0.01), with absolute volumes of 125.63 ± 111.92 cc and 149.53 ± 116.16 cc. For V_11.6Gy_, the relative volumes were 8.82 ± 8.18% for iBT and 10.13 ± 7.61% for SBRT (p < 0.01), with corresponding absolute volumes of 116.69 ± 104.47 cc and 136.78 cc ± 105.99 cc. The relative differences in median values between HDR-iBT and SBRT were − 5.29% for V_5Gy_ (range: −17.89% to + 13.69%), −1.51% for V_9.1Gy_ (−12.56% to + 6.25%), −1.51% for V_10Gy_ (−11.26% to + 7.74%), −1.12% for V_11Gy_ (−9.69% to + 8.79%), and − 0.95% for V_11.6Gy_ (−9.15% to + 8.86%). Despite these differences, all liver–GTV dose parameters stayed within established dose constraints in both SBRT and iBT plans; only one case of liver constraint violation occurred in a plan involving 5 GTVs (Table 2 and Fig. 3, Fig. 4, Fig. 5).

No significant differences were observed for other non-critical organs at risk (Supplementary Table 1), and no notable differences were found between SBRT and iBT plans with respect to other OAR constraints.

## Discussion

To the best of our knowledge, this is the first comparative planning study evaluating the plan quality and dosimetric properties of delivered brachytherapy versus *in silico* planned MRL-based single-fraction SBRT in colorectal liver metastases.

Both HDR-iBT and *in silico* SBRT achieved excellent target volume coverage, with a mean GTV V_25Gy_ of approximately 100% in both instances, inferring effective delivery of the prescribed doses. The mean GTV D_98%_ values were comparable between HDR-iBT (28.82 ± 2.57 Gy) and SBRT (28.92 ± 0.88 Gy), indicating comparable gross tumor volume coverage. While HDR-iBT demonstrated a slightly higher mean GTV D_95%_ (31.62 ± 3.20 Gy) compared to SBRT plans (29.22 ± 0.74 Gy, p < 0.01), SBRT exhibited more consistent dosimetric reliability, with a narrower standard deviation and a tighter dose range compared to HDR-iBT. HDR-iBT demonstrated a significant advantage in dose intensification to the tumor core, as indicated by higher GTV D_50%_ values (64.7 vs. 30.2 Gy).

A strength of the current analysis is the use of clinical cases of HDR-iBT instead of simulated plans. Single-fraction SBRT improves convenience by reducing treatment time and avoiding session interruptions, while maintaining comparable toxicity through precise targeting and elimination of interfractional variability [[14], [15], [16], [17]]. Ahmed et al. reported on the radiosensitivity index in metastatic liver lesions and noted differences in the index based on histology, with colorectal adenocarcinoma among the more radioresistant histologies [18]. There is, however, a rationale for higher dose SF-SBRT or multi-fraction SBRT. Recently, we published the results of SF-SBRT (28–30 Gy, prescribed to the 80% IDL) in lung metastases, and interestingly, 3/4 local failures (LFs) were attributed to colorectal histology [19]. The Stanford Group recently published the results of personalized SBRT for lung tumors. This trial treated tumors with individually dosed and fractionated SBRT (NCT01463423), based on tumor volume, location, and histology. Peripheral non-colorectal tumors ≤ 10 cc were treated with a single fraction of 25 Gy prescribed to cover 95% of the PTV, while colorectal cancer (CRC) metastases received 50 Gy in 4 fractions. This individualized treatment approach resulted in excellent local control rates, with 1-year LC ranging from 94% to 97% and 5-year LC from 83% to 93%, depending on the patient subgroup, and was associated with low toxicity. Notably, no local failures were observed in the subgroup of 17 patients with colorectal histology who were treated with high-dose, multi-fraction SBRT [20]. However, with high-dose irradiation (approximately ≥ 10 Gy/fraction), there may be multiple mechanisms affecting tumor cell survival, such as damage to tumor vasculature and antitumor immunity, that are difficult to model [21].

While single fraction dose delivery is an intriguing proposition, a German Society of Radiation Oncology multicenter database modeling analysis revealed a maximum biologically effective dose (BED_max_) of 257 ± 74 Gy without prior chemotherapy (corresponding to a clinical dose prescription of, for example, 3 x 16 Gy to the 65% IDL) and 335 ± 73 Gy with prior chemotherapy (corresponding to a clinical dose prescription of, for example, 3 x 19 Gy to the 65% IDL) to achieve at least 90% local control at 2 years for CRLM [22]. This BED_max_ cannot be achieved by a physical dose prescription of 25 Gy to the 80% IDL. However, such doses are easily attainable with brachytherapy. Meyer JJ et al. established 35–40 Gy SF-SBRT, prescribed to the 60–90% IDL for selected patients with liver metastasis [23]. A recently published single-arm phase 2 trial by Chuong et al., investigating MR-guided SF-SBRT in multiple locations, prescribed a dose of 35–40 Gy to liver metastases, with hotspots of at least 120–130% of the prescribed dose encouraged, corresponding to a prescription to approximately the 80% IDL [24].

Several studies have examined planning coverage and dosimetric characteristics of brachytherapy and SBRT [5,[25], [26], [27]]. In our previous study, we analyzed liver exposure differences between single-session brachytherapy (1 x 15 Gy) and fractionated SBRT (3 x 12.5 Gy prescribed to the 65% IDL) for 71 hepatocellular carcinoma lesions [5]. The current study directly compares single-session brachytherapy with single-fraction SBRT, offering a more reliable assessment by eliminating uncertainties vis-à-vis the biological effects of fractionation.

Studies have shown that the outcome of SBRT depends on the delivered dose and the extent of tumor coverage [1,14,17,23,28]. Goodman et al. conducted a phase I dose escalation study of single-fraction SBRT for liver lesions, including those from CRC, where single doses ranging from 18 Gy to 30 Gy were applied. The mean dose to the uninvolved liver was 8 Gy or less for all patients. This approach showed promising local control with minimal acute and long-term toxicity, and no patients experienced dose-limiting toxicity [14]. Lanciano et al. reported that liver metastases treated with a BED greater than 100 Gy achieved better local control, with a rate of 75% at 2 years, compared to only 38% for those receiving less than 100 Gy [29]. Similarly, Doi et al. demonstrated that higher doses, particularly those exceeding a BED of 100 Gy, were associated with improved outcomes; however in multivariate analysis only smaller tumors (≤ 30 mm) remained significantly associated with superior local control [30]. Stinauer et al. found that patients receiving 45 Gy or more as a single-fraction equivalent dose for radiation-resistant metastases other than colorectal carcinoma achieved a 100% local control rate at 24 months, vs. 54% for those under 45 Gy [31]. In another study involving patients with liver metastases from colorectal cancer treated with SBRT, those who received a BED_10_ of 100–112 Gy demonstrated significantly improved local control, with a reported hazard ratio of 0.44 [32]. While further research and clinical trials may refine these results, current evidence strongly supports using higher peripheral doses, greater than 28–30 Gy, in liver SBRT.

Treatment-related toxicity plays a critical role in determining therapeutic outcomes for patients with liver tumors [33]. In a study on liver tumors, higher liver doses were strongly associated with liver function decline following SBRT [34]. Son et al. reported that the progression of Child-Pugh class was significantly associated with the liver volume receiving greater than 18 Gy [35]. Therefore, minimizing radiation dose to uninvolved liver tissue is essential, especially for patients with limited hepatic reserve. In our analysis, a median relative difference of 5.29% was observed for uninvolved liver V_5Gy_, with SBRT consistently delivering slightly higher relative volumes for other dose metrics, including V_9.1Gy_ and V_11Gy_ (median differences of 1.51% and 1.12%). Although statistically significant, these constraints remained within clinically acceptable limits. A previous study by our group demonstrated that the average liver volume exposed to 10 Gy in a single fraction of HDR-iBT (V_10Gy_) was smaller than the corresponding volume exposed to 20 Gy in multi-fraction SBRT [5]. In a similar comparative analysis, Hass et al. evaluated iBT and *in silico* SBRT plans for liver malignancies, reporting significantly lower liver dose exposure with iBT-findings that are consistent with our results [26]. The ability of HDR-iBT to deliver lower doses to uninvolved liver tissue makes it particularly advantageous for patients with limited hepatic reserve or those at increased risk for liver toxicity.

No significant differences were observed in organ-at-risk dose constraints for non-critical structures since iBT, while feasible, was not delivered to the central liver near the hilum/porta hepatis. No significant difference was observed in doses delivered to the gastrointestinal luminal organs. However, in MRL-based planning for centrally located liver lesions, daily adaptive planning can reduce radiation to sensitive gastrointestinal luminal organs [7]. Typically, organ at risk constraints take priority over tumor coverage, using a strict isotoxicity approach. The prescribed dose relies on a planning target volume optimization structure, defined as the PTV minus the OAR plus a 3 mm margin. This method allows maximum ablative dose delivery to the tumor while managing OAR doses based on daily position, rather than static constraints used outside adaptive radiation therapy. Research shows that small, reduced doses in the overlap between PTV and OARs do not significantly impact local control [36].

Regarding practical implementation, CT-guided HDR interstitial brachytherapy provides highly conformal dose delivery and is particularly useful in anatomically complex or previously irradiated regions. However, its use can be logistically and clinically challenging in patients on anticoagulation therapy or those at higher risk for complications such as abscess formation. MR-guided radiotherapy enables real-time soft-tissue visualization and daily adaptive planning, offering precise targeting for lesions near critical structures, though its use may be limited by prolonged treatment times, patient tolerance issues (e.g., claustrophobia, need for extended immobilization), and restricted availability of MR-linac platforms. CT-guided online adaptive SBRT has evolved into a practical alternative, integrating daily image guidance and plan adaptation to account for anatomical variability [[37], [38], [39], [40]].

A brief planning report of the first 60 patients who underwent radiation planning for the SABR-COMET-10 randomized trial, assessing the effect of SBRT in patients with a controlled primary and 4–10 metastatic lesions, was previously published. Of the 332 lesions treated, 26 (7.8%) were located in the liver [41]. This ongoing study evaluates the feasibility of multi-organ, multi-lesion SBRT.

## Limitations

The retrospective nature of the study introduces potential biases in patient selection and data interpretation. The small sample size, comprising only 26 patients and 45 lesions, limits the generalizability of the results to larger and more diverse populations. Additionally, treatment plans were transferred from the Oncentra system to the ViewRay TPS platform, which may have introduced discrepancies in structure volumes due to voxelization, resolution differences, or variations in DICOM structure interpolation between the two systems. In order to mitigate this issue, we also reported relative differences between both modalities.

## Conclusion

This study provides the first direct comparison between single-fraction HDR brachytherapy and MRL-based SBRT for colorectal liver metastases. Both techniques demonstrated excellent target volume coverage, with comparable GTV D_98%_ values and adherence to organ-at-risk dose constraints. HDR-iBT showed a clear advantage in dose escalation within the tumor core, as reflected by higher GTV D_1cc_ and D_50%_ values, while *in silico* SBRT plans demonstrated greater consistency and minimal variability. Although uninvolved liver dose exposure was slightly higher with SBRT plans, it remained within acceptable clinical limits.

The real-time adaptability and precision of MRgSBRT underscore its potential for broader clinical application, warranting further investigation in prospective clinical trials.

## CRediT authorship contribution statement

**Sina Mansoorian:** Data curation, Formal analysis, Methodology, Validation, Visualization, Writing – original draft, Writing – review & editing. **Svenja Hering:** Data curation, Formal analysis, Methodology, Validation, Visualization, Writing – review & editing. **Jan Hofmaier:** Formal analysis, Methodology, Validation, Visualization, Writing – review & editing. **Yuqing Xiong:** Formal analysis, Methodology, Validation, Visualization, Writing – review & editing. **Helmut Weingandt:** Formal analysis, Methodology, Validation, Visualization, Writing – review & editing. **Maya Rottler:** Data curation, Formal analysis, Methodology, Validation, Visualization, Writing – review & editing. **Franziska Walter:** Data curation, Formal analysis, Methodology, Validation, Visualization, Writing – review & editing. **Paul Rogowski:** Conceptualization, Formal analysis, Methodology, Validation, Writing – review & editing. **Max Seidensticker:** Conceptualization, Formal analysis, Methodology, Validation, Writing – review & editing. **Jens Ricke:** Conceptualization, Formal analysis, Methodology, Validation, Writing – review & editing. **Claus Belka:** Conceptualization, Formal analysis, Methodology, Validation, Writing – review & editing, Funding acquisition, Investigation, Project administration, Supervision. **Stefanie Corradini:** Conceptualization, Formal analysis, Methodology, Validation, Writing – review & editing, Funding acquisition, Investigation, Project administration, Supervision. **Chukwuka Eze:** Conceptualization, Data curation, Formal analysis, Funding acquisition, Methodology, Supervision, Visualization, Writing – original draft, Writing – review & editing.

## Declaration of Competing Interest

The authors declare that they have no known competing financial interests or personal relationships that could have appeared to influence the work reported in this paper.
