# Supplementary material for: Comparative analysis of target volume coverage and liver exposure in high-dose-rate interstitial brachytherapy and in silico MR LINAC-based stereotactic body radiotherapy plans for colorectal liver metastases
Source: Clin Transl Radiat Oncol. 2025 Aug 11;56:101030. doi: 10.1016/j.ctro.2025.101030 (PMC12454280; doi:10.1016/j.ctro.2025.101030)
Supplement: Supplementary Data 1 [file mmc1.docx]

**Supplementary Table 1** Dose-volume parameters for other organs at risk in iBT vs. SBRT, showing no significant differences between the two planning modalities (N.A. = Not Applicable); only provided for cases with at least 5 pairs

| Organ | constraint | n values pairs | p-value (Wilcoxon) | constraint | n values pairs | p-value (Wilcoxon) |
| --- | --- | --- | --- | --- | --- | --- |
| Stomach | <1 cc at 12 Gy | 5 | 0.625 | <0.1 cc at 14 Gy | 5 | 0.625 |
| Colon | <1 cc at 14 Gy | 5 | 0.250 | <0.1 cc at 18 Gy | 4 | N.A. |
| Heart | <1 cc at 20 Gy | 7 | 0.250 | D_max_ < 22 Gy | 6 | 0.125 |
| Kidney | V7Gy < 66% | 5 | 0.813 |  |  |  |

**Supplementary Table 2:** Summary of GTV Dose Coverage in 45 Paired SBRT and iBT Plans

| Parameter | SBRT (n = 45) | iBT (n = 45) |
| --- | --- | --- |
| Cases with D_100%_ ≥ Prescription Dose | 42 (93.33%) | 28 (62.22%) |
| Cases with D_100%_ < Prescription Dose | 3 (6.67%) | 17 (37.78%) |
| Cases where SBRT had better D_100%_ | 43 (95.56%) | – |
| Cases where SBRT had worse D_100%_ | 2 (4.44%) | – |
| V_25Gy_ = 100% | 42 (93.33%) | 39 (86.67%) |
| V_25Gy_ < 100% | 3 (6.67%) | 6 (13.33%) |
| Range of V_25Gy_ when <100% | 99.40%–99.93% | 99.00%–99.99% |


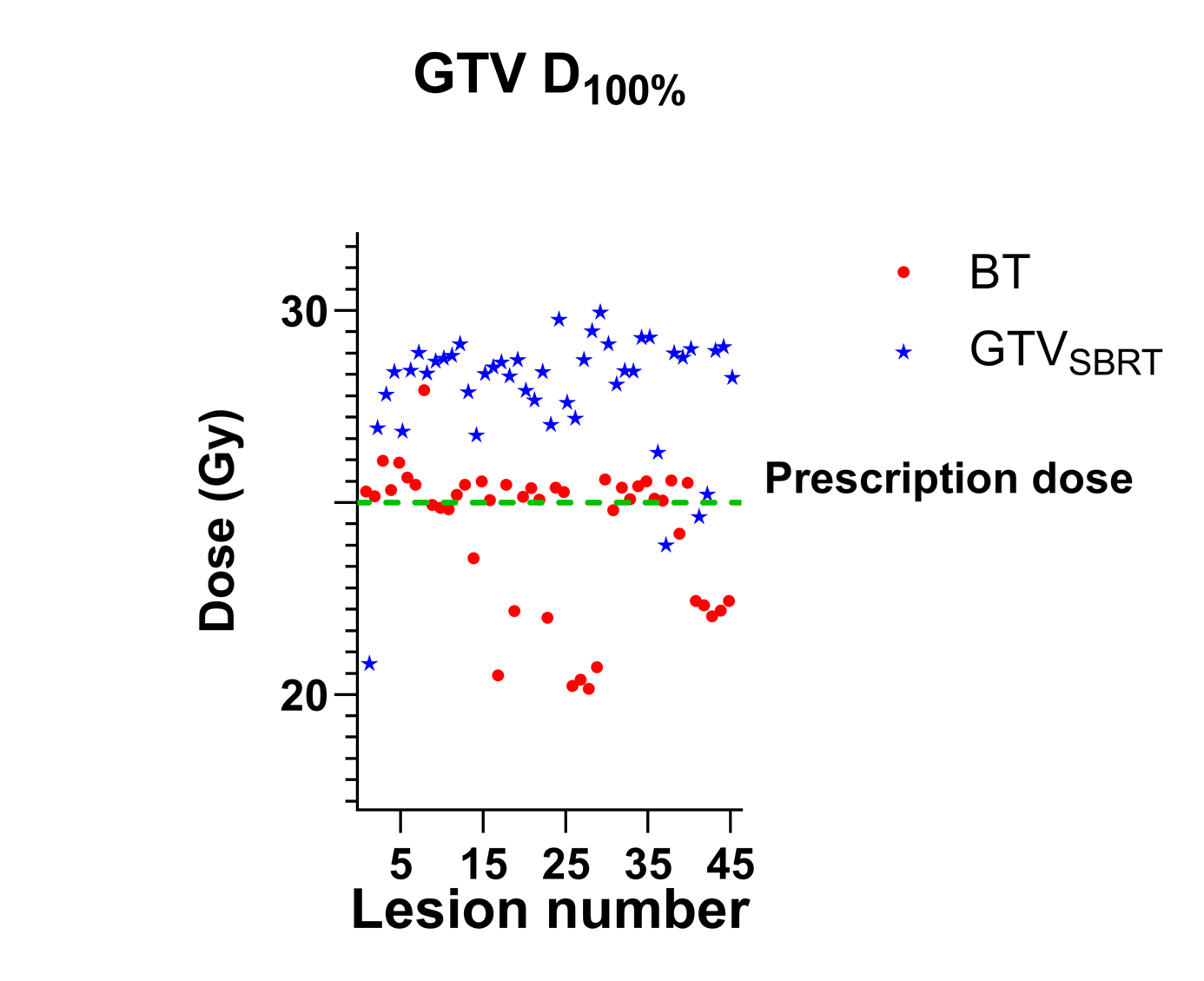


Supplementary Figure 1 Comparison of D_100%_ values between SBRT (blue stars) and iBT (red circles) across 45 paired treatment plans. Each pair represents a single case, illustrating the consistently higher minimum dose coverage (D_100%_) achieved with SBRT in most cases.
